# Manipulation of magnetic vortex parameters in disk-on-disk nanostructures with various geometry

**DOI:** 10.3762/bjnano.6.70

**Published:** 2015-03-10

**Authors:** Maxim E Stebliy, Alexander G Kolesnikov, Alexey V Ognev, Alexander S Samardak, Ludmila A Chebotkevich

**Affiliations:** 1 Laboratory of Thin Film Technologies, School of Natural Sciences, Far Eastern Federal University, Vladivostok 690950, Russia

**Keywords:** hysteresis, magnetic vortex, magnetization reversal, micromagnetic structure

## Abstract

Magnetic nanostructures in the form of a sandwich consisting of two permalloy (Py) disks with diameters of 600 and 200 nm separated by a nonmagnetic interlayer are studied. Magnetization reversal of the disk-on-disk nanostructures depends on the distance between centers of the small and big disks and on orientation of an external magnetic field applied during measurements. It is found that manipulation of the magnetic vortex chirality and the trajectory of the vortex core in the big disk is only possible in asymmetric nanostructures. Experimentally studied peculiarities of a motion path of the vortex core and vortex parameters by the magneto-optical Kerr effect (MOKE) magnetometer are supported by the magnetic force microscopy imaging and micromagnetic simulations.

## Introduction

Magnetic nanostructures have a wide range of unique properties that facilitate their practical implementation in real functional devices. For instance, magnetoresistive memory is used in aviation systems requiring the high reliability, nonvolatility and data write/read rate. Nanomagnets with stable and controllable spin configurations can be used to produce the magnetic logic elements. However, micromagnetic stability decreases with the reduction of the size of a nanomagnet resulting in a lack of controllability the spin configurations [1]. Therefore, the development of reliable methods for the manipulation of micromagnetic structures in nanomagnets is an important topic not only for fundamental physics, but mostly for high-tech sectors of economics including electronics, data storage and sensor technologies.

Nanomagnets in the shape of disks attract huge scientific attention, because of the possibility to realize the four stable magnetic vortex states with different combinations of polarity and chirality [2]. Polarity (up or down out-of-plane component of magnetization in the central core of the vortex state) can be controlled by an external magnetic field aligned perpendicular to a disk plane. This method is complicated to be used for microelectronic applications due to the high value of an applied field (usually, larger than 1 kOe [3]).

The in-plane component of the vortex state is characterized by the clockwise (CW) or counterclockwise (CCW) magnetization rotation or chirality. The majority of methods of chirality manipulation are based on an application of magnetic asymmetry in disks through cutting of a part of the disk, forming of a hole or producing a magnetization gradient [4–7]. However, the modified shape or magnetization asymmetry of a disk results in significant changes in the values of vortex nucleation and annihilation fields. Moreover, the trajectory of a vortex core is distorted and its gyrotropic motion frequency changes significantly.

Recently, by using magnetic force microscopy (MFM), we have demonstrated a reliable method to control the vortex parameters in a big disk if a disk with smaller diameter is placed on its top [8]. In this paper, we present results of the impact of the small disk position relative to a symmetry axis of the “small disk on big disk” nanostructure. By using a measurement method based on the magneto-optical Kerr effect (MOKE), the vortex chirality in disks has been reliably defined. Peculiarities of the vortex nucleation process as well as the vortex core trajectory under an impact of bias fields have been observed. Experimental findings have been interpreted by micromagnetic simulations [9].

## Results and Discussion

**Figure 1:**
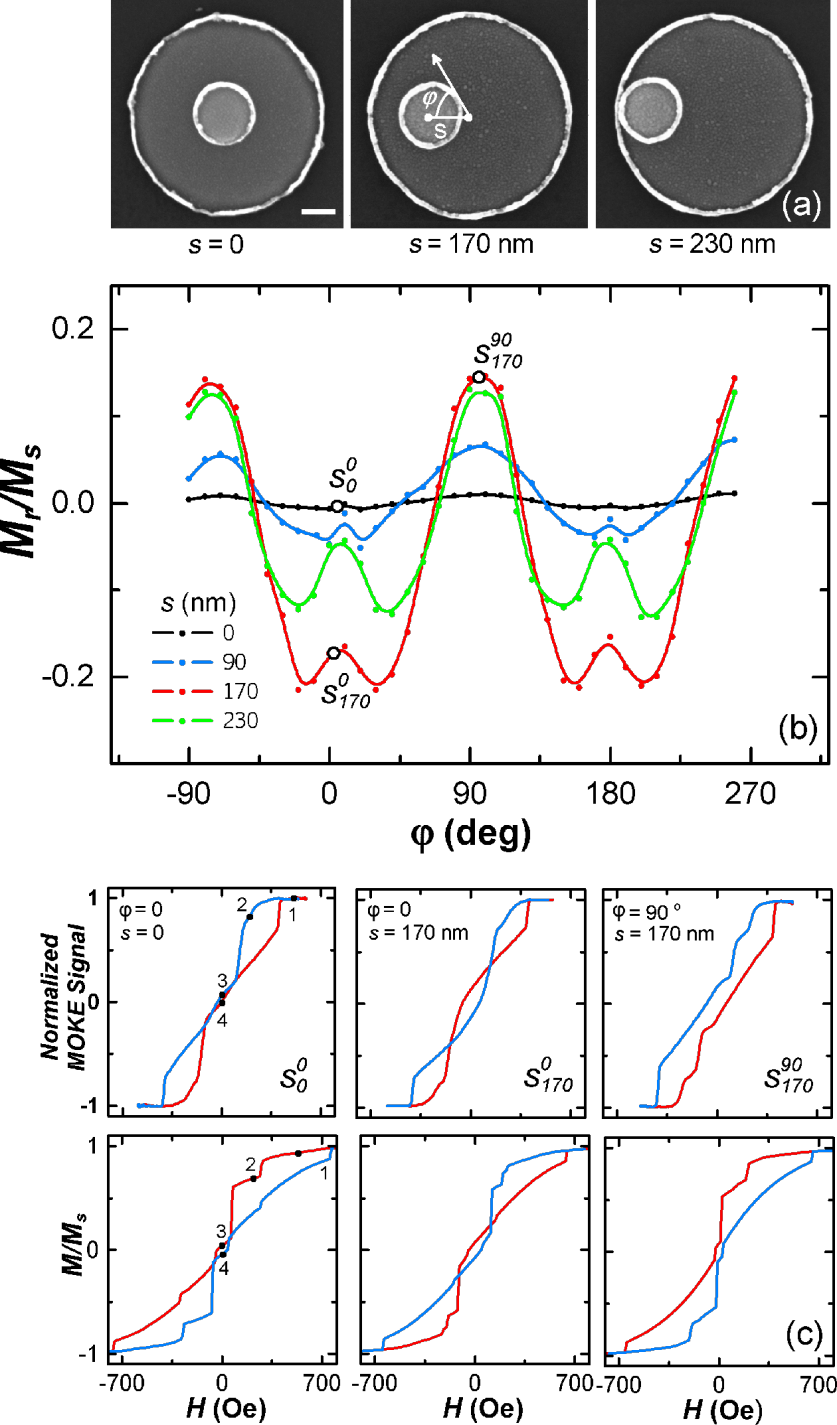
(a) SEM images of the disk-on-disk nanostructure with *s* = 0, 170 and 230 nm; (b) Dependence of *M*_r_/*M*_s_ on the magnetic field orientation relatively to the *x*-axis; (c) experimental (upper row) and simulated (bottom row) hysteresis loops for following cases: (i) s = 0 and φ = 0, (ii) s = 170 nm and φ = 0, (iii) s = 170 nm and φ = 90^o^.

Figure 1a shows scanning electron microscopy (SEM) images of disk-on-disk nanostructures with different distances between its centers, *s*, which range from 0 (small disk in the center) to 230 nm (small disk at the edge of big disk). We have experimentally studied the effect of the orientation of the disk-on-disk nanostructure relatively to an external magnetic field on the magnetization reversal in dependence on *s*. The angle φ, defining the orientation of an applied magnetic field, was counted from the selected *x*-axis, passing through the centers of the disks. The results are shown in Figure 1b. It was found that the magnetization reversal changes qualitatively. For symmetric nanostructures with *s* = 0 the *M*–*H* curve is anhysteretic (Figure 1c, loop 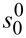) and its shape does not depend on the orientation of the external field (Figure 1b). In asymmetric nanostructures (s ≠ 0) the angular dependence is strongly uniaxial with the maxima at 90° and −90°: *M*_r_/*M*_s_ > 0 (where *M*_r_ is the remanence and *M*_s_ is the saturation magnetization) and the hysteresis loop is open (Figure 1c, loop 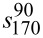). There are two local maxima at φ = 0 and 180° surrounded by two minima, Figure 1b. In the field, aligned at an angles 0 and 180°, the hysteresis loop has an inverted shape, i.e., *M*_r_/*M*_s_ < 0. The maximum change of *M*_r_/*M*_s_ was observed in nanostructures with *s* = 170 nm.

To understand the physics of magnetization reversal in disk-on-disk nanostructures, micromagnetic simulations and MFM measurements were conducted. Calculated hysteresis loops are shown in the bottom row of Figure 1c. As can be observed seen, the shapes of the loops, as well as the critical fields, are in a good agreement with the experimental data presented in the upper row of Figure 1c. There are several areas with a sharp jump in magnetization at values of *H* of about 150 and 230 Oe.

### Magnetic properties of symmetric nanostructures

**Figure 2:**
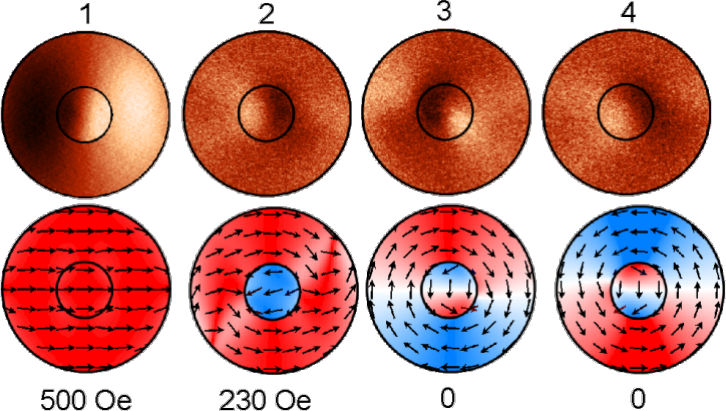
MFM images of magnetic structure (upper row) and corresponding spin configurations (bottom row) in the field oriented at φ = 0.

Let us consider the magnetization reversal in a case when *s* = 0. Figure 2 shows the results of MFM study and calculated spin configurations at magnetic fields marked with numbers in the hysteresis loop (Figure 1c, loop 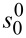). With the reduction of the external field from the positive saturation, *H*_s_ (point 1), the disk-on-disk system turns from a homogeneous magnetic state into the state with antiparallel magnetization due to the magnetization switching in the small disk (point 2). As can be observed, there is a step in the hysteresis loop. With further decrease of the field the magnetic vortex emerges in the big disk, meanwhile the “C-shape” state is formed in the small disk (point 3). If the external field is increased from negative saturation, −H_s_, the magnetization reversal does not change qualitatively, but at *H* = 0 (point 4 in Figure 1c) the magnetization in the small disk becomes opposite to the field orientation. Vortex chirality switches from CW to CCW direction (Figure 2, case 4). There is an intercoupling between the vortex chirality in the big disk and the magnetization in the small disk. If in the small disk the “C-shape” state has CCW chirality, than the big disk will have CW chirality, and vice versa. Thus, applying a positive or negative field along φ = 0, one could control the vortex chirality in the big disk. However, in a symmetric disk-on-disk system the formation of “C-shape” state in the small disk and the vortex nucleation in the big disk are equally probable. Therefore, it is impossible to reliably control the chirality in the big disk.

### Magnetic properties of asymmetric nanostructures

In the case of asymmetric nanostructures with *s* ≠ 0, the magnetization reversal depends on the orientation of the field relatively to the direction of *s*. (Figure 1c, loops 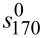 and 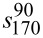). The MFM study showed that the magnetization reversal is similar to the processes in a symmetric structure: The single-domain state forms in the small disk, and the vortex forms in the big disk. However, there are some features caused by asymmetry.

### Remagnetization in the magnetic field at φ ≈ 0

**Figure 3:**
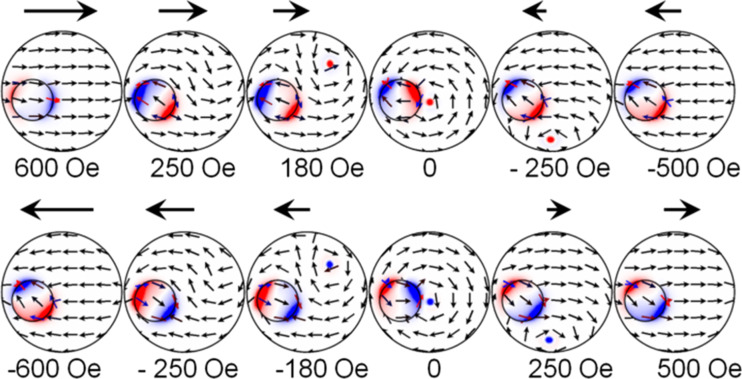
Spin configurations realized in the disk-on-disk nanostructure in dependence on an external magnetic field H applied at φ ≈ 0.

Let us consider the case in which the field *H*_x_ is applied at an angle φ ≈ 0, i.e., directed along the selected *x*-axis. MFM contrast, formed by the single-domain state of the small disk, dominates over the weak contrast of the vortex state in the big disk. Therefore, to analyze the MFM data, micromagnetic simulations of spin configurations were used (Figure 3). At saturation (*H*_s_ > 500 Oe) disks are in single-domain states with collinear orientation of magnetization. At a field of H ≈ 230 Oe the small disk is in single-domain state with the magnetization, oriented antiparallel to the magnetization in the big disk. In the field H ≈ 150 Oe the vortex nucleates at the edge of the big disk. With the decrease of the field down to zero the vortex core is shifted to the center of the disk. With the rise of negative fields, the vortex core is shifted to the lower edge of the disk and then it annihilates.

If the magnetization reversal of the disk-on-disk nanostructure in the fields ranging from −*H*_s_ to +*H*_s_ is analyzed, the quality of the reversal process does not change. But at *H* = 0, the magnetization in the small disk is oriented in the direction opposite to the field. The chirality of the vortex is reversed and becomes rotated clockwise. The asymmetric structure enables one to control the magnetic vortex chirality in the big disk through the orientation of the magnetic field. How to use the magneto-optical Kerr effect to define the magnetic vortex chirality in the disk-on-disk nanostructure will be discussed below.

**Figure 4:**
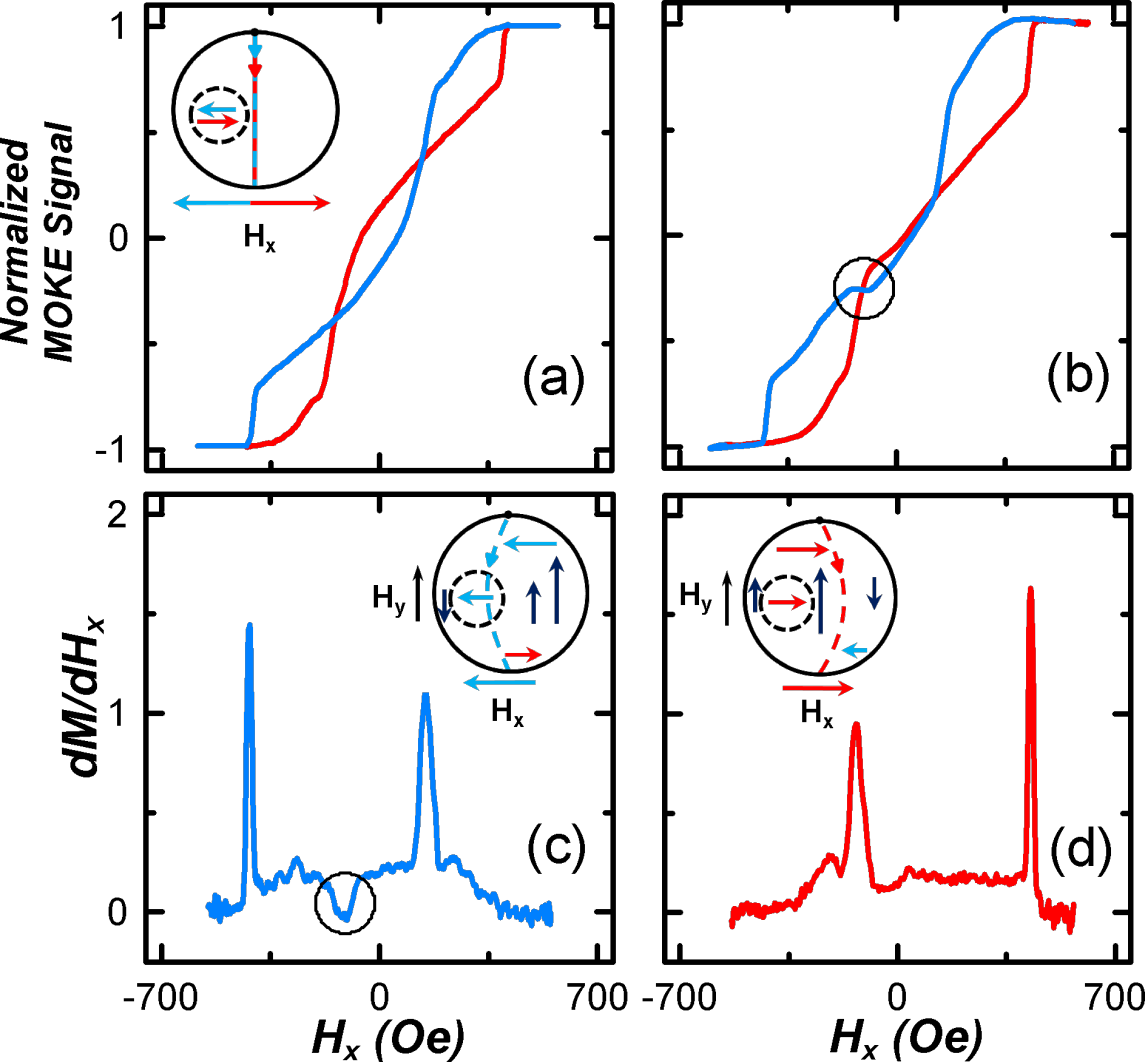
Hysteresis loops corresponding to the magnetization reversal of the disk-on-disk nanostructure without (a) and with bias field *H*_y_ (b). (c,d) The first derivative curves *dM/dH_x_* defined for different branches of the hysteresis loop with bias field *H_y_*. Insets show the trajectory of the vortex core motion with the magnetization reversal without (a) and with bias field *H_y_* (c,d). Insets illustrate the schematic view of trajectories of the vortex core in dependence on the applied magnetic fields.

According to the micromagnetic simulations, the vortex nucleates in the same place of the big disk, regardless of the orientation of *H_x_*. Then the vortex core along a path that does not intersect the small disk (see inset in Figure 4a). As can be observed, there are no steps in the hysteresis loop due to the magnetostatic interaction of the small disk and the vortex core. Applying the constant magnetic field *H*_y_ perpendicular to the *s* direction (i.e., along the *y*-axis), the vortex-core trajectory changes. The direction of displacement of the vortex core depends on its chirality. The outline of this process is shown in the inset of Figure 4c and Figure 4d. In the case of CCW chirality (Figure 4c), the vortex core is deflected to the small disk. Passing under the small disk, the vortex core interacts with it. This affects the magnetic hysteresis loop in which a characteristic step appears. If the chirality has CW rotation, the vortex core is deflected to the right side without any interaction with the small disk (Figure 4d).

Figure 4 shows the magnetic hysteresis loops measured only in the field *H_x_* (Figure 4a) and in the presence of a bias field *H_y_* (Figure 4b). As can be observed, the loop becomes asymmetric under impact of *H_y_*. During magnetization reversal in the fields from +*H*_s_ to −*H*_s_ (blue curve) ranging from −86 to −146 Oe, a typical step, characterized by a constant magnetization in dependence on the field magnitude, appears in the loop. In the *dM*/*dH_х_* curve, shown in Figure 4c, the change of the magnetization velocity is zero matching the pinning of the vortex core by the small disk (marked by the circle). The shifted trajectory of the vortex core is shown in the inset in Figure 4c. There is no step on the return curve (when the field changing from −*H*_s_ to +*H*_s_) due to a vortex core pinning, because the core is moving away from the small disk (see the inset in Figure 4d). The velocity of the vortex in the fields ranging from 20 to 350 Oe is almost constant (Figure 4d). The preservation of the loop asymmetry during multiple magnetization reversals shows that if the field is changed from +*H*_s_ to −*H*_s_, the vortex chirality exhibits only CCW rotation, and from −*H*_s_ to +*H*_s_ the chirality is rotated clockwise.

### Remagnetization in magnetic field at φ ≈ 90°

**Figure 5:**
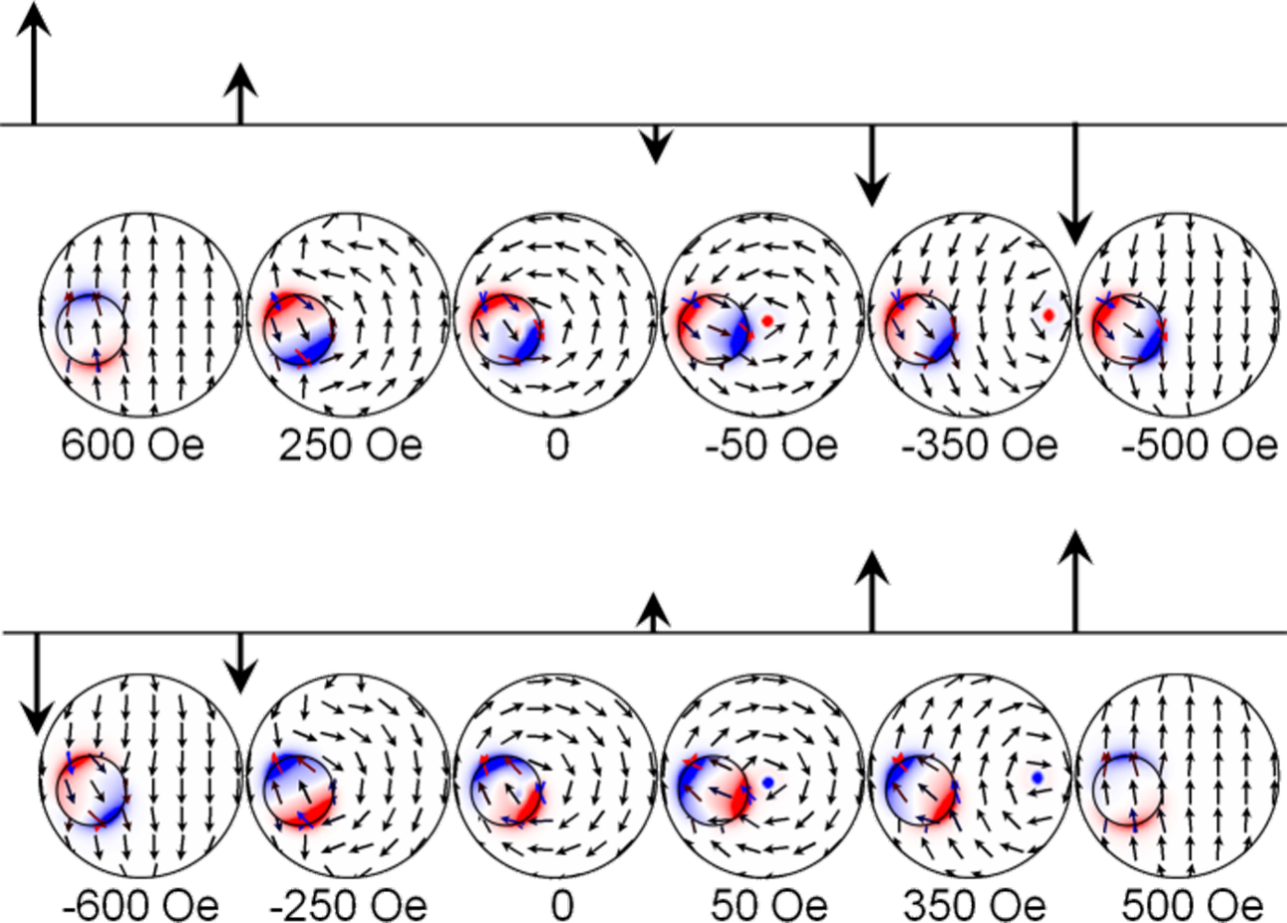
Spin configurations in the disk-on-disk nanostructure during the magnetization reversal under the applied magnetic field *H* oriented at φ ≈ 90°.

Let us consider a case in which the magnetic field is oriented perpendicular to the *s*-direction, i.e., φ ≈ 90°. MOKE results and calculated loops for this case are shown in Figure 1c. Micromagnetic simulations (Figure 5) and MFM data point out that the magnetizations of the small and big disks at saturation are oriented along the applied field direction. With the field is reduced down to 300 Oe, the single-domain state switches in the small disk leading to an antiparallel alignment of the magnetic moments in adjacent layers (AP state). This change of the magnetic state causes the first step in the hysteresis loop. Then, the vortex with CCW chirality nucleates in the big disk from the small disk side. When the magnetic field is decreased to zero, the vortex core is shifted to the center of the small disk. This is reflected in a relative small change of the magnetization in the field ranging from *H*_n_ to 0. At *H* = 0 the vortex core is shifted from the disk center, so the remanence is positive (no inversion as in the previous case). In the negative fields the vortex core gets out from under the small disk, moves through the center of the disk to the edge and then annihilates.

When the magnetization reversal occurs under a field between *−H*_s_ and 0, the magnetization of the small disk is directed opposite to the field. The vortex in the big disk also nucleates from the side of the small disk, but the vortex chirality in the big disk changes and the magnetization vector rotates clockwise. Thus, because of the asymmetry of the magnetization distribution in the nanostructure, the vortex chirality depends on the direction of an applied magnetic field, and the vortex nucleation always occurs on the side of the small disk and does not depend on the field orientation.

**Figure 6:**
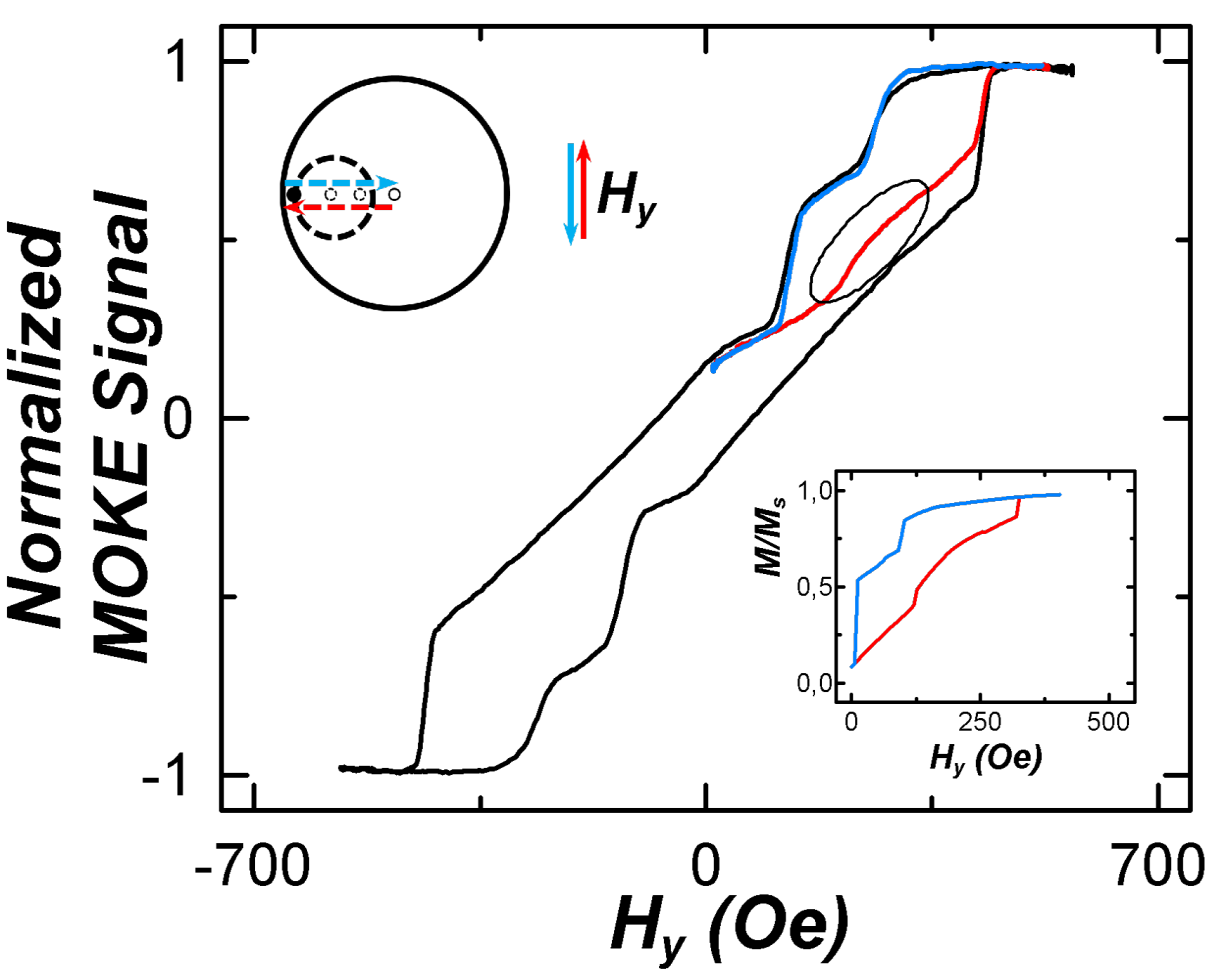
Hysteresis loops measured under a field ranging from +500 to −500 Oe (black line), as well as under an asymmetric field changing from +500 to −50 Oe (colored lines). Insets represent the schematic view of trajectories of the vortex core (top) and the result of micromagnetic simulations of the hysteresis loop (bottom).

For experimental verification of the vortex chirality and trajectory of the core movement, MOKE measurements were used. The results are shown in Figure 6. The local magnetization loop in the field ranging from +500 to −50 Oe was measured. The *M*/*M*_s_ curve (blue line) in the field ranging from +500 to −50 Oe matches the full hysteresis loop (black line). The reverse curve (red line) in the range from −50 to +500 Oe differs from the reverse branch of the hysteresis loop.

Results of the micromagnetic simulations show that in fields changing from +500 to −50 Oe, the transition of the spin configuration is the same as in Figure 5. Vortex nucleates under the small disk with the following core movement to the center of the big disk (blue arrow in the upper insert in Figure 6). When the field switches to the opposite direction (from −50 to +500 Oe), the core is moving to the nucleation site where the vortex annihilates (red arrow in the upper inset in Figure 6). Passing under the small disk, the vortex core interacts with its magnetic poles. This is reflected in the simulated hysteresis loop in the form of a step (red curve in the lower inset in the Figure 6). This step is seen in the experimental local loop (marked by the oval on the red curve). A similar dependence is observed when the field is changing from −500 to 50 Oe.

Thus, when the field *H* is directed at φ ≈ 90°, the vortex nucleates only under the small disk, and the orientation of the single-domain state in the small disk and the vortex chirality in the big disk depend on the direction of the external magnetic field [8].

It is clear that one can control the vortex chirality in the big disk by changing the direction of an external magnetic field. However, it is crucial to the break the symmetry of the system with respect to chirality. That is why in case of symmetric nanostructures the chirality control is impossible. In the asymmetric nanostructure, the inhomogeneous magnetostatic field is induced between the small disk in single-domain state or “C-shape” state and the big disk. This leads to a breaking of the circular symmetry and, consequently, gives an opportunity to control the chirality and nucleation point of the vortex.

**Figure 7:**
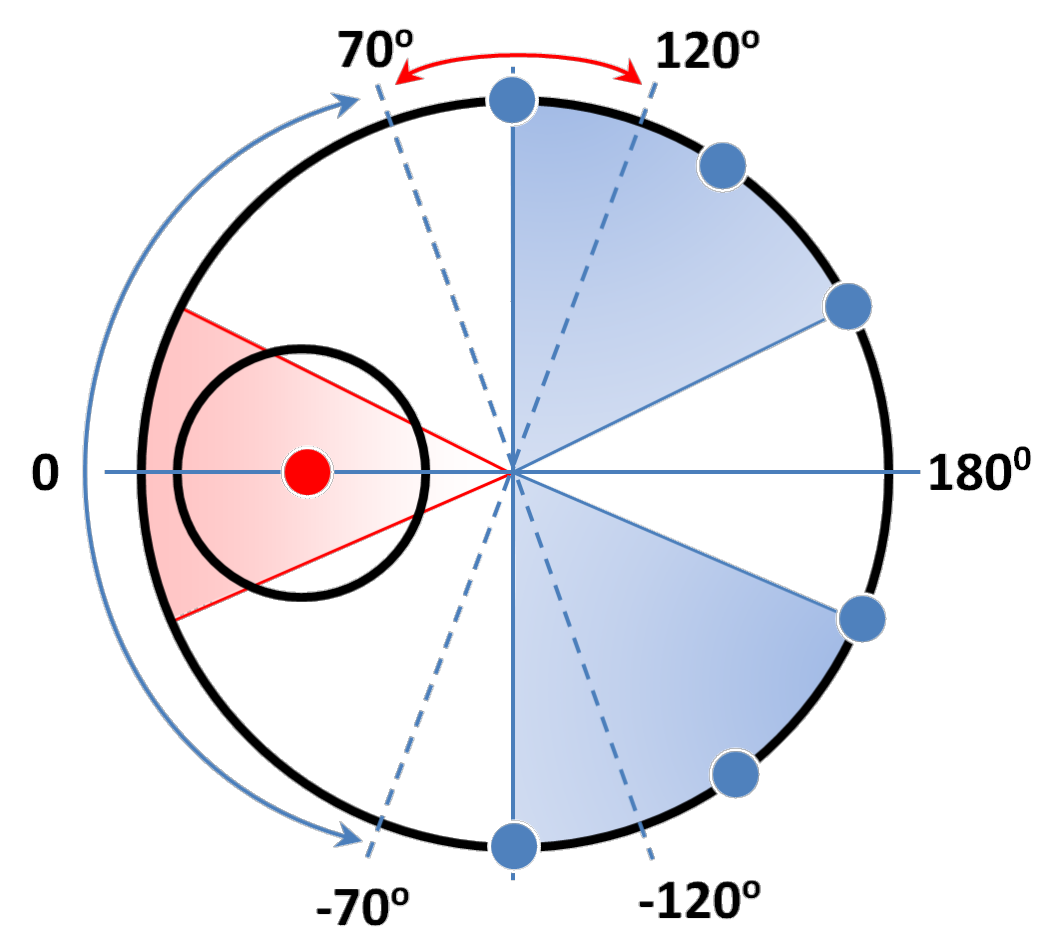
Scheme explaining the interplay between the orientation of an external magnetic field (angles are marked by the blue and red arrows) and location of the vortex nucleation sites in the big disk (blue and red points).

Figure 7 shows the scheme of the location of vortex nucleation sites depending on the direction of an external field for nanostructures with *s* = 170 nm. To construct the scheme the experimental data *M*_r_/*M*_s_ = f(φ) (see Figure 1a), and the micromagnetic simulation results were used. When *M*_r_/*M*_s_ < 0, the magnetic field is applied under an angle from 70 to −70° (indicated by the blue arrow in Figure 7) and the vortex nucleation sites are located in the blue sectors, being perpendicular to the field orientation (marked with blue points in the scheme). Here, for a vortex nucleation in the upper and lower sectors, the field *H* has to be directed at φ > 0 and φ < 0, correspondingly. As seen in Figure 3, the nanostructure is slightly turned at the angle φ = 5° and the vortex nucleates in the upper sector. As for this direction of the field the vortex nucleation site is constant, the variation of the field from +*H*_s_ to 0 or from 0 to −*H*_s_ leads to a controlled change of the vortex chirality: CCW or CW, respectively. If apply the field is applied at φ = 0, which is difficult to implement experimentally, the probability of vortex nucleation in the upper and lower sectors is equal and the chirality control fails.

If *M*_r_/*M*_s_ > 0, the field has to be applied at an angle between 70 and 120° (the range indicated by the red arrow in Figure 7). In this case, the vortex nucleation site is located only in the red sector. The micromagnetic calculations show that for the given configuration of the magnetic field, the vortex nucleation is energetically favorable under the small disk (marked with the red point in the scheme). This fact has been confirmed experimentally by MOKE measurements (see Figure 6). To control the chirality of the vortex, as in the case discussed above, the field has to be applied at an angle different from 90^o^. For example, Figure 5 shows the simulation results for the case in which *H* is oriented at φ = 95°. Then for a magnetization reversal from +*H*_s_ to 0, the vortex has a CCW chirality, and from −*H*_s_ to 0 it has CW chirality.

## Conclusion

The magnetic behavior of disk-on-disk nanostructures with various geometries is demonstrated. The magnetization reversal of disk nanostructures depends on the distance between the centers of the small and big disks as well as on the orientation of an applied magnetic field. The inverted character of hysteresis loops in the case of an asymmetric geometry of the disk-on disk nanostructures was found. Maximum inversion of the remanence value was observed for *s* = 170 nm. It is shown that at remanence the big disk has the vortex configuration, meanwhile the small disk is in the single-domain state. The reliable control of spin configurations in the disks at remanence can be uniquely specified by the orientation of the external magnetic field. The formation of stable vortex configurations with a desired chirality was experimentally demonstrated. Our study shows that the vortex chirality in the asymmetric disk-on-disk nanostructures can be explicitly defined by MOKE measurements. Our findings open new opportunities for practical implementation of the vortex-based devices such as memory cells [10] and ternary logic elements [11].

## Experimental

The experimental study was conducted for disk-on-disk nanostructures consisting of two permalloy (Py or Ni_80_Fe_20_) nanodisks with a thickness of 35 nm. The big (bottom) and small (upper) disks with diameters of 600 and 200 nm, respectively, were separated by a 3 nm thick Cu interlayer. The nanostructures were fabricated on naturally oxidized Si(111) substrates by means of electron-beam lithography, magnetron sputtering and standard lift-off process. Geometry and surface roughness were checked with scanning electron (SEM, Supra, Carl Zeiss) and atomic force (AFM, Ntegra Aura, NT-MDT) microscopes. Magnetic properties were studied by using a magneto-optical Kerr effect (MOKE, NanoMOKE II) magnetometer and a magnetic force microscope (MFM, Ntegra Aura, NT-MDT). Micromagnetic simulations were performed by using OOMMF software [9] with standard parameters for Py: *M*_s_ = 860 Gs, exchange stiffness *A* = 1.38 · 10^6^ erg/cm, damping factor α = 0.05 [11]. The magnetic anisotropy was chosen zero in order not to insert an asymmetry of magnetic properties into the system. Dimension of the simulated disk-on-disk nanostructure matched the experimental sample. Cell size was taken as 3 × 3 × 35 nm^3^.

## References

[1] Coffey W T, Kalmykov Y P (2012). J Appl Phys.

[2] Guslienko K Yu, Novosad V, Otani Y, Shima H, Fukamichi K (2001). Phys Rev B.

[3] Kikuchi N, Okamoto S, Kitakami O, Shimada Y, Kim S G, Otani Y, Fukamichi K (2001). J Appl Phys.

[4] Zhong Z, Zhang H, Tang X, Jing Y, Jia L, Liu S (2009). J Magn Magn Mater.

[5] Dumas R K, Gredig T, Li C-P, Schuller I K, Liu K (2009). Phys Rev B.

[6] Cambel V, Karapetrov G (2011). Phys Rev B.

[7] Liu Y, Du A (2010). J Appl Phys.

[8] Stebliy M E, Ognev A V, Samardak A S, Diga K S, Chebotkevich L A (2012). IEEE Trans Magn.

[9] (2014). OOMMF Project at NIST.

[10] Kim S-K, Lee K-S, Yu Y-S, Choi Y-S (2008). Appl Phys Lett.

[11] Stebliy M E, Ognev A V, Samardak A S, Kolesnikov A G, Chebotkevich L A, Han X (2014). Appl Phys Lett.

